# Mesenchymal stem cell treatment for chronic renal failure

**DOI:** 10.1186/scrt472

**Published:** 2014-07-04

**Authors:** Alfonso Eirin, Lilach O Lerman

**Affiliations:** 1 Division of Nephrology and Hypertension, Mayo Clinic, 200 First Street SW, Rochester, MN 55905, USA

## Abstract

Chronic renal failure is an important clinical problem with significant socioeconomic impact worldwide. Despite advances in renal replacement therapies and organ transplantation, poor quality of life for dialysis patients and long transplant waiting lists remain major concerns for nephrologists treating this condition. There is therefore a pressing need for novel therapies to promote renal cellular repair and tissue remodeling. Over the past decade, advances in the field of regenerative medicine allowed development of cell therapies suitable for kidney repair. Mesenchymal stem cells (MSCs) are undifferentiated cells that possess immunomodulatory and tissue trophic properties and the ability to differentiate into multiple cell types. Studies in animal models of chronic renal failure have uncovered a unique potential of these cells for improving function and regenerating the damaged kidney. Nevertheless, several limitations pertaining to inadequate engraftment, difficulty to monitor, and untoward effects of MSCs remain to be addressed. Adverse effects observed following intravascular administration of MSCs include immune rejection, adipogenic differentiation, malignant transformation, and prothrombotic events. Nonetheless, most studies indicate a remarkable capability of MSCs to achieve kidney repair. This review summarizes the regenerative potential of MSCs to provide functional recovery from renal failure, focusing on their application and the current challenges facing clinical translation.

## Introduction

Chronic kidney disease (CKD) is a prevalent condition (8 to 16%) associated with all-cause and cardiovascular mortality [1]. Importantly, CKD can progress towards end-stage renal disease (ESRD), requiring renal replacement therapy. ESRD currently accounts for 6.3% of the Medicare spending in the United States, and is projected to increase by 85% by 2015 [2]. Furthermore, ESRD has a tremendous impact on quality of life and life expectancy of affected individuals [3]. Therefore, it is imperative to develop therapeutic interventions to prevent, alleviate, or decelerate progression of renal failure.

Diabetes mellitus and hypertension represent major causes of CKD and initiation of dialysis in the United States [4]. In addition, glomerular diseases, malnutrition, infectious diseases, and acute kidney injury can progress to ESRD, contributing to the increased global burden of death associated with this condition [5]. Current treatment modalities often fail to target the major underlying contributors for progression of renal disease [6]. Chronic glomerular and tubulointerstitial fibrosis is a common pathway to ESRD, often associated with apoptosis, oxidative damage, and microvascular rarefaction. In fact, renal dysfunction is postulated to better correlate with the degree of tubulointerstitial than with glomerular damage [7].

Importantly, the kidney possesses intrinsic regenerative capacity that allows the organ to recover after limited insults [8]. Unfortunately, this regenerative potential is limited under chronic conditions and thus inefficient to prevent progressive glomerulosclerosis and tubulointerstitial fibrosis [9]. Treatment strategies that boost cellular regeneration might therefore offer good alternatives for patients with CKD.

Mesenchymal stem cells (MSCs) can be isolated from a variety of tissues, differentiate into multiple cell lineages, and possess unique immunomodulatory properties that ameliorate inflammation and immune responses, constituting a promising tool to facilitate renal repair. MSCs are defined by the presence of plastic-adherent cells under standard culture conditions, capacity to differentiate into osteoblasts, adipocytes and chondroblasts *in vitro*, expression of typical surface markers (CD29, CD44, CD73, CD90, CD105, and CD166), and the lack of CD45, CD34, CD14 or CD11b, CD79α or CD19 and HLA-DR surface molecules [10]. In recent years, experimental studies have uncovered the potential of MSCs to improve renal function in several models of CKD, and several clinical studies have indicated their safety and efficacy in CKD. Nevertheless, a number of hurdles need to be addressed before clinical translation. This review summarizes the current state of MSC transplantation for CKD, focusing on their mechanisms of renal repair, complications, obstacles for clinical translation, and potential approaches to overcome them.

## Mesenchymal stem cells in experimental chronic kidney disease

Over the past few years, MSCs have been successfully applied in experimental models of CKD such as diabetes, hypertension, and chronic allograft nephropathy (Table 1). For example, a single intravenous dose of MSCs resulted in beta-pancreatic islet regeneration, prevented renal damage in streptozotocin-induced type 1 diabetes in C57BL/6 mice [11], and decreased hyperglycemia and glycosuria that persisted for 2 months after injection. Furthermore, MSC-treated diabetic mice showed histologically normal glomeruli, and albuminuria fell. Although the authors did not assess cellular mechanisms associated with MSC therapeutic effects, the long-lasting persistence of injected MSCs may suggest a direct effect to elicit kidney regeneration.

**Table 1:** Preclinical studies using mesenchymal stem cells for the treatment of chronic kidney disease

**Disease**	**Source**	**Dose**	**Route**	**Mechanism of action**	**Side effects**	**Reference**
Diabetic nephropathy	Mice bone marrow	0.5 × 10^6^	Intravenous	Engraftment/direct effect	None	[11]
Diabetic nephropathy	Human bone marrow	2 × 10^6^	Intracardiac	Engraftment/direct effect	None	[12]
Partial nephrectomy	Rat bone marrow	1 × 10^6^	Intravenous	Paracrine effect	None	[13]
Chronic allograft nephropathy	Rat bone marrow	0.5 × 10^6^	Intravenous	Immunomodulatory effect	None	[14]
Renal revascularization	Allogeneic swine adipose tissue	10 × 10^6^	Intrarenal	Engraftment/direct effect/paracrine	None	[16,17]
Renal artery stenosis	Autologous swine adipose tissue	10 × 10^6^	Intrarenal	Engraftment/direct effect/paracrine	None	[15]

Similarly, Lee and colleagues tested the effectiveness of intracardiac infusions of MSCs from human bone marrow in immunodeficient mice with type 2 diabetes produced with multiple low doses of streptozotocin [12]. MSCs lowered blood glucose levels and decreased mesangial thickening and macrophage infiltration, suggesting their potential for improving renal lesions in subjects with diabetes mellitus. Interestingly, in kidneys of MSC-treated diabetic mice, a few injected human MSCs differentiated into glomerular endothelial cells.

Additionally, in rats with modified 5/6 nephrectomy, a single venous injection of MSCs 1 day after nephrectomy preserved renal function and attenuated renal injury [13]. Despite unchanged blood urea nitrogen and creatinine levels, MSC-treated animals showed attenuated progression of proteinuria. The scarce engraftment of MSCs in the kidneys of rats with chronic renal failure suggests that paracrine secretion of mediators, such as cytokines or growth factors, may have accounted for their beneficial effects. Indeed, vascular endothelial growth factor (VEGF) levels were substantially higher in MSC-treated animals 1 month after MSC injection.

Furthermore, a single dose of bone marrow-derived MSCs 11 weeks after kidney transplantation in rats decreased interstitial fibrosis, tubular atrophy, T-cell and macrophage infiltration, and the expression of inflammatory cytokines [14]. Interestingly, a decrease over time in the inflammatory and profibrotic cytokine levels in MSC-treated animals was associated with an increase in the anti-inflammatory cytokine IL-10, although none of the injected MSCs were detected 7 days after delivery. These observations imply that the beneficial effect of these cells in this setting is primarily attributable to their paracrine immunomodulatory properties rather than long-term engraftment.

**Figure 1:**
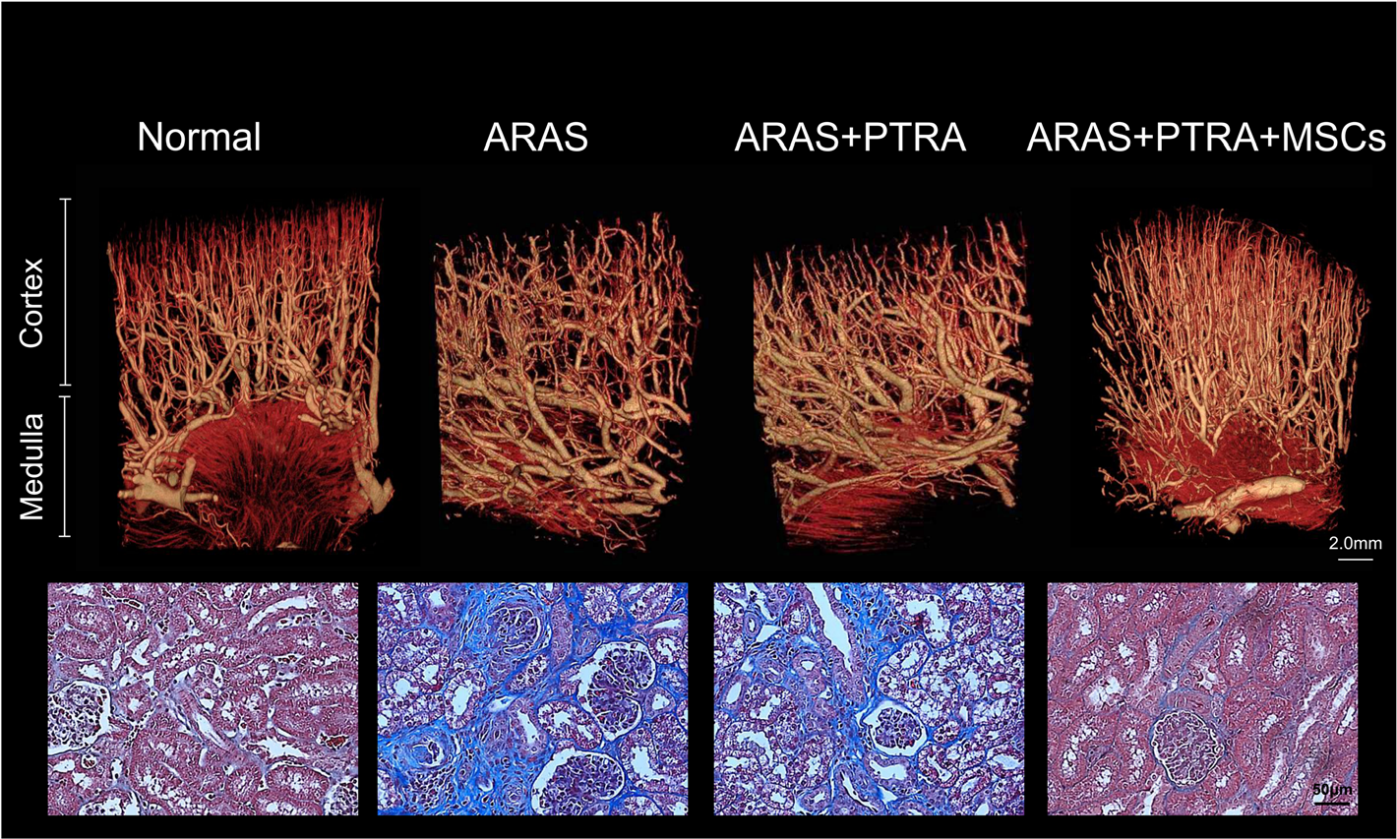
**Stenotic-kidney microvascular loss and fibrosis decreased in animals treated with mesenchymal stem cells.** Top: representative microcomputed tomography three-dimensional images of kidney segments, showing improved microvascular architecture in pigs with atherosclerotic renal artery stenosis (ARAS) treated with percutaneous transluminal renal angioplasty (PTRA) and an adjunct intrarenal infusion of adipose tissue-derived mesenchymal stem cells (MSC) 4 weeks earlier. Bottom: representative renal trichrome staining (×40, blue) showing decreased fibrosis in ARAS + PTRA + MSC pigs.

We have previously shown in swine atherosclerotic renovascular disease that intrarenal delivery of MSCs isolated from subcutaneous adipose tissue protected the stenotic kidney despite sustained hypertension [15]. Notably, MSCs also attenuated renal inflammation, endoplasmic-reticulum stress, and apoptosis through mechanisms involving cell contact. Furthermore, adjunctive MSCs improved renal function and structure after renal revascularization and reduced inflammation, oxidative stress, apoptosis, microvascular remodeling, and fibrosis in the stenotic kidney [16] (Figure 1). This strategy also restores oxygen-dependent tubular function in the stenotic-kidney medulla, extending their value to preserving medullary structure and function in chronic ischemic conditions [17].

## Potential challenges for clinical translation

While preclinical studies have established the safety and efficacy of MSCs in different models of CKD, these results need cautious translation into routine clinical practice. Trials using MSCs for CKD patients may face various challenges, including selecting the optimal route of MSC delivery, scant homing and engraftment, immune rejection, ensuring thriving, and tracking of injected cells. Addressing these challenges may bolster the success of MSC therapy in improving renal function in CKD patients.

### Route of delivery

The route of MSC delivery may influence the cells’ capacity to home and engraft the damaged tissue, and thereby their efficacy for renal repair. Commonly used experimental methods to deliver MSCs include systemic intravenous, intra-arterial, or intraparenchymal delivery. When intravenously delivered in normal Sprague–Dawley rats, the majority of MSCs are initially trapped in the lungs [18], but in nonhuman primates the cells distribute broadly into the kidneys, skin, lung, thymus, and liver with estimated levels of engraftment ranging from 0.1 to 2.7% [19]. In contrast, direct delivery of MSCs into the renal artery of an ischemic kidney is associated with retention rates of 10 to 15% [16,17], although the normal swine kidney retains only around 4%, due to the low tonic release of injury signals. However, injection of human MSCs into the mouse abdominal aorta may lead to occlusion in the distal vasculature due to their relatively large cell size (16 to 53 μm), suggesting that this approach should be used cautiously [20]. Injections of MSCs into the renal parenchyma or their local subcapsular implantation confer renoprotective effects [21,22], but are difficult to implement in the human injured kidney.

In experimental models of CKD, the optimal dose of MSCs is often empirical, with doses ranging from 0.5 × 10^6^ to 10 × 10^6^[11,16]. Despite variability in dose regimens and route of delivery, the safety and beneficial effects of MSCs were consistent among studies. Nevertheless, the use of escalating doses is strongly recommended in clinical trials, and chronic adverse events should be evaluated prior to enrollment at the next dose level.

### Homing

Circulating hematopoietic progenitor cells home to the damaged kidney by responding to injury signals that correspond to cognate surface receptors which they express [23]. Accumulating evidence indicates that exogenously infused MSCs respond to similar homing signals. In mice, expression of CD44 and its major ligand hyaluronic acid mediates MSC migration to the injured kidney [24], and hyaluronic acid also promotes MSC dose-dependent migration *in vitro*. Moreover, renal homing of intravenously injected MSCs was blocked by preincubation with the CD44 blocking antibody or by soluble hyaluronic acid, suggesting that CD44 and hyaluronic acid interactions recruit exogenous MSCs to the injured kidney. In addition, Liu and colleagues found that, when administered systemically, MSCs home to the ischemic kidney, improving renal function, accelerating mitogenic response, and reducing cell apoptosis, but these effects were abolished by either CXCR4 or CXCR7 inhibition, implicating the stromal derived factor-1–CXCR4/CXCR7 axis in kidney repair [25].

Collectively, these observations suggest that strategies aimed to enhance MSC expression of homing signals may improve their capacity to attenuate renal dysfunction. Studies have shown that selective manipulation of MSCs before transplantation (preconditioning) enhances their ability to protect damaged tissues [26,27]. The rationale underpinning this approach is that transplanted MSCs encounter a hostile microenvironment that mitigates their reparative capabilities and survival. Indeed, preconditioning with the mitogenic and prosurvival factor insulin-like growth factor (IGF)-1 before systemic infusion of bone marrow-derived MSCs (2 × 10^5^) upregulates the expression of CXCR4 and restores normal renal function in a mice model of cisplatin-induced acute kidney injury [28].

### Engraftment

Some studies suggest that MSCs have the capacity to engraft the damaged tissue, integrate into tubular cells, and differentiate into mesangial cells [29-31]. In swine renovascular disease, 4 weeks after intrarenal infusion, MSCs (10 × 10^6^) were detected in all regions of the kidney, but mostly at the renal interstitium [16,17]. On the other hand, intravenous infusion of bone marrow-derived MSCs (2 × 10^5^) in mice with cisplatin-induced acute renal failure reduced the severity of renal injury, but none were detected within the renal tubules and only few cells within the renal interstitium at 1 to 4 days after infusion [32], suggesting that MSC engraftment is not necessary to achieve renoprotection. Likewise, despite significant improvement in renal function, within 3 days of intracarotid infusion in a rat model of ischemia–reperfusion-induced acute renal failure, none of the MSCs differentiated into the tubular or endothelial cell phenotype, indicating that their beneficial effects are primarily mediated via paracrine actions rather than differentiation into target cells [33].

Methods to increase MSC engraftment may therefore enhance their utility in regenerative cellular therapy. Temporary obstruction of the renal artery following intrarenal delivery [16,17] may prevent cell washout, and is associated with significant retention rates in the postischemic kidney. Alternatively, in a rat model of acute kidney injury, s-nitroso *N*-acetyl penicillamine preconditioning enhances MSC engraftment, ultimately associated with a significant improvement in renal function [34].

Despite the crucial role attributed to MSC engraftment in potentiating the cells’ beneficial effect at the site of injury, there is currently consensus that the chief mechanism by which MSCs protect the damaged kidney is the release of growth factors, proangiogenic factors, and anti-inflammatory cytokines. Cultured MSCs release large amounts of the proangiogenic factor VEGF, which facilitates glomerular and tubular recovery [16,35]. MSCs can also produce IGF-1, while administration of IGF-1 gene-silenced MSCs limits their protective effect on renal function and tubular structure in murine cisplatin-induced kidney injury, indicating that MSCs exert their beneficial effects by producing IGF-1 [36].

Importantly, these paracrine actions of MSCs seem to mediate their immunomodulatory properties. In ischemia–reperfusion-induced acute kidney injury, infusion of MSCs downregulates renal expression of proinflammatory cytokines and adhesion molecules such as IL-1β, tumor necrosis factor alpha, interferon gamma, monocyte chemoattractant protein-1, and intercellular adhesion molecule-1, but upregulates the expression of the anti-inflammatory IL-10 [26,33]. Likewise, we have shown in swine renovascular disease that intrarenal delivery of MSCs during renal revascularization decreased renal expression of tumor necrosis factor alpha and monocyte chemoattractant protein-1, but increased IL-10 expression [17]. Moreover, MSCs induced a shift in the macrophage phenotype from inflammatory (M1) to reparative (M2), uncovering their immunomodulatory potential [37]. Taken together, these observations underscore the contribution of paracrine actions of MSCs to induce a shift from an inflammatory to an anti-inflammatory microenvironment. It is not unlikely that the type, number, and expansion methods used to secure MSCs alter their engraftment capacity.

### Rejection

For many years, MSCs have been considered immune privileged because of the lack of expression of co-stimulatory molecules and their capacity to decrease renal release and expression of inflammatory mediators [17,33,37]. These attributes engendered the hope that MSCs could engraft in allogeneic nonimmunosuppressed recipients, and stimulated development of off-the-shelf allogeneic MSC products [38], which allow rapid generation of large amounts of cells from few donors. Nevertheless, *in vivo* and *in vitro* studies have demonstrated that MSCs may occasionally induce an immune switch transitioning from an immunoprivileged to an immunogenic phenotype that triggered cellular cytotoxicity or immune rejection [39]. Moreover, implantation of murine MSCs engineered to release erythropoietin in major histocompatibility complex-mismatched allogeneic mice increased the proportion of host-derived lymphoid CD8^+^ and natural killer infiltrating cells, suggesting that MSCs are not intrinsically immunoprivileged [40]. Taken together, these observations do not support the use of allogeneic MSCs as a universal cellular platform, at least until development of unequivocally immunoprivileged MSCs. Therefore, at this point, administration of autologous MSCs seems to be the safest strategy.

### Thriving

An important feature of MSCs is their capacity to induce proliferation of renal glomerular and tubular cells, increasing cellular survival. By secreting proangiogenic and trophic factors, injected MSCs not only can enhance proliferation, but also can decrease apoptosis of tubular cells [32]. We have shown in swine renovascular disease that a single intrarenal delivery of MSCs in conjunction with renal revascularization increased proliferation of renal cells [16], and recently confirmed *in vitro* that MSCs blunt apoptosis by decreasing the expression of caspase-3 [15].

However, whether MSCs remain in the circulation long enough to exert any long-lasting effect is a matter of debate. Ezquer and colleagues showed that intravenous MSCs home into the kidney of type 1 diabetic mice, and some donor MSCs remained in the kidney up to 2 months later [11]. Similarly, we found that 4 weeks after intrarenal delivery a significant number of MSCs were retained in the injected kidney [16,17], whereas by 12 weeks after cell transfer only a few cells were observed in the kidney, yet their beneficial effects were sustained [15]. Longitudinal studies are needed to document the chronology of MSC retention and beneficial benefits in the kidney. Additionally, development of novel interventions such as preconditioning may enhance survival and potency of MSCs in renal failure. For instance, MSCs exposed to hypoxic conditions in culture sustain viability and function through preservation of oxidant status [41], and preconditioning with kallikrein [26] or melatonin [27] enhances their therapeutic potential.

An important challenge for clinical translation is the risk for long-term MSC maldifferentiation. While intrarenal injection of rat MSCs initially preserves renal function in a rat model of glomerulonephritis, a significant proportion of the glomeruli subsequently contained large adipocytes with glomerular sclerosis [42]. Furthermore, reports of sarcoma [43] and teratoma [44] arising from exogenous MSCs illustrate their potential for transformation into tumors, underscoring the requirement for closely monitoring human MSCs in clinical studies. Alternatively, complications and maldifferentiation of live replicating MSCs warrant development of safer tactics and interventions.

**Figure 2:**
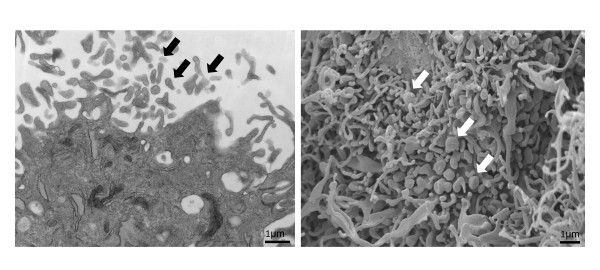
**Mesenchymal stem cell release microvesicles.** Transmission electron microscopy image (left) and scanning electron microscopy image (right) showing release of microvesicles (arrows) from adipose tissue-derived mesenchymal stem cells (×26,500).

Considerable evidence shows that MSCs release microvesicles which exhibit characteristics of their parental cells, and transfer proteins, lipids, and genetic material to target cells. We have recently shown that endothelial outgrowth cells release microvesicles [45], which may mediate their intercellular communications. Similarly, MSCs are avid producers of microvesicles [46] (Figure 2) that shuttle functional components for their paracrine action [47]. Delivery of microvesicles instead of their parent MSCs could avoid concerns about extensive expansion, cryopreservation, complications, and maldifferentiation of live replicating cells. Indeed, microvesicles derived from preconditioned MSCs promoted recovery in a rat hind-limb ischemia model [48]. However, questions regarding their composition and potency relative to their parent MSCs remain unanswered, underscoring the need for studies to clarify the potential of this promising therapeutic modality.

Uremic conditions may also affect the efficacy of MSCs, limiting their potential use in patients with CKD. Uremia induced by partial kidney ablation in C57Bl/6 J mice leads to MSC functional incompetence, characterized by decreased expression of VEGF, VEGF receptor-1, and stromal derived factor-1, increased cellular senescence, and decreased proliferation [49]. Conversely, MSCs isolated from subcutaneous adipose tissue of healthy controls and patients with renal disease show similar characteristics and functionality, underscoring the feasibility of autologous cell therapy in patients with renal disease [50]. Indeed, a recent meta-analysis of prospective clinical trials that used intravascular delivery of MSCs concluded that these cells have an excellent safety record [51].

### Interspecies differences in the biology of mesenchymal stem cells

Although it is accepted that MSCs from different species are capable of differentiation into various lineages and express common MSC markers, species-dependent variability in their expression has been reported among different species [52]. Furthermore, the mechanism of MSC-mediated immunosuppression varies among different species. For example, while immunosuppression by human-derived or monkey-derived MSCs is mediated by indoleamine 2,3-dioxygenase, the molecular mechanisms underlying immunosuppression in mouse MSCs utilize nitric oxide [53]. Several immune barriers have been also encountered in experimental xenotransplantation, the transplantation of MSCs from one species to another, warranting the development of genetic alternatives to overcome these obstacles [54]. Clearly, results from experimental studies need to be carefully validated before clinical translation.

### Tracking

There is also a pressing need for better methods for detection and monitoring the fate of MSCs. Despite improvement in direct (fluorescent probe) [55] and indirect (reporter genes) [56] labeling techniques, questions regarding interactions of MSCs with tissue, differentiation, or migration remain unanswered. While fluorescent probes such as membrane tracers or microspheres need to be detected with histological techniques in a cell or organelle, reporter genes such as bioluminescence or fluorescent proteins can be used to identify different cell populations using imaging *in vivo*[57,58]. However, these detection methods have little tissue penetration, limiting their use in large animal models or humans [59].

Conceivably, imaging modalities such as single-photon emission computed tomography or magnetic resonance imaging may address some of these deficiencies by providing high-resolution anatomical detail and tracking of cell viability [60,61]. Several types of agents are currently used for labeling MSCs for their detection with magnetic resonance imaging. Among them, superparamagnetic iron oxide particles are the most commonly applied, because of their capacity to induce changes in T2 relaxivity *in vivo*[62]. However, the transfection agents used for superparamagnetic iron oxide particle internalization may also affect cell viability, and dying cells accumulate iron until dissolved or eliminated by phagocytosis, impeding their application as indices of cell viability. Further methods are therefore needed to better assess engraftment, survival, and function of MSCs in human subjects.

## Clinical trials using mesenchymal stem cells for renal repair

Few clinical trials have tested safety and efficacy of MSCs for renal disease. Reinders and colleagues studied safety and feasibility in six kidney allograft recipients who received two intravenous infusions of expanded autologous bone marrow-derived MSCs (10^6^ cells/kg, 7 days apart) because of rejection and/or increased interstitial fibrosis and tubular atrophy [63]. Although the design of the study does not allow one to draw conclusions on efficacy, in two recipients with allograft rejection the renal biopsies after MSC treatment demonstrated resolution of tubulitis without interstitial fibrosis and tubular atrophy, whereas maintenance immune suppression remained unaltered, supporting the potential of MSCs in preventing allograft rejection. However, three patients developed an opportunistic infection, raising concerns regarding systemic immunosuppression after MSC infusions. Similarly, a recent prospective, open-label, randomized study demonstrated that, among patients undergoing renal transplant, intravenous infusion of marrow-derived autologous MSCs (1 × 10^6^ to 2 × 10^6^/kg) at kidney reperfusion and 2 weeks later decreased the incidence of acute rejection and of opportunistic infection, and improved renal function at 1 year compared with anti-IL-2 receptor antibody induction therapy [64]. Importantly, delivery of autologous MSCs was not associated with adverse events, nor did it compromise graft survival. Likewise, autologous MSC infusion in two recipients of kidneys from living-related donors 7 days post-transplant restricted memory T-cell expansion and enlarged the T-regulatory cell population [65]. These observations suggest safety and clinical feasibility of cell-based therapy with MSCs in the context of kidney transplantation.

Several clinical trials are currently underway to evaluate the therapeutic potential of autologous and allogeneic MSCs for treatment of renal diseases [66]. For example, NCT01843387 investigates the safety, tolerability and efficacy of a single intravenous infusion of two doses of MSCs versus placebo in subjects with diabetic nephropathy and type 2 diabetes. NCT00659620 will test whether MSCs are effective in preventing organ rejection and maintaining kidney function in patients who develop chronic allograft nephropathy. In addition, NCT00659217 evaluates infusion of expanded autologous MSCs into patients with lupus nephritis. Finally, NCT01840540 is a phase I study of autologous MSCs in the treatment of atherosclerotic renal artery stenosis. While they aim primarily to test the feasibility and practical usefulness of MSCs in renal diseases, results from these clinical trials may also shed light on the mechanisms responsible for MSC renal protection.

## Conclusions and future perspectives

Available experimental evidence confirms that MSCs contribute to cellular repair and ameliorate renal injury in CKD. Although a few safety and feasibility clinical studies suggest their capacity to repair the damaged kidney, several barriers need to be circumvented in order to consider MSCs as a realistic clinical tool to treat CKD. Among them, systemic immunosuppression and adipogenic/malignant transformation raise major concerns. Additionally, the route of MSC delivery and complexity of CKD patients (for example, uremia) should be considered when designing clinical studies. Development of novel therapies such as microvesicles and preconditioning might promote engraftment and MSC communication with injured parenchymal cells. Further large controlled clinical trials are needed to assess the efficacy and safety profile of MSCs in CKD.

## Note

This article is part of a thematic series on *Stem cells in genitourinary regeneration* edited by John Jackson. Other articles in the series can be found online at http://stemcellres.com/ series/genitourinary

## Abbreviations

CKD: Chronic kidney disease; ESRD: End-stage renal disease; IGF: Insulin-like growth factor; IL: Interleukin; MSC: Mesenchymal stem cell; VEGF: Vascular endothelial growth factor.

## Competing interests

The authors declare that they have no competing interests.
